# Modulation of Brain Resting-State Networks by Sad Mood Induction

**DOI:** 10.1371/journal.pone.0001794

**Published:** 2008-03-19

**Authors:** Ben J. Harrison, Jesus Pujol, Hector Ortiz, Alex Fornito, Christos Pantelis, Murat Yücel

**Affiliations:** 1 Melbourne Neuropsychiatry Centre, Department of Psychiatry, The University of Melbourne and Melbourne Health, Victoria, Australia; 2 Institut d'Alta Tecnologia-PRBB, CRC Corporació Sanitària, Barcelona, Spain; 3 ORYGEN Research Centre, Melbourne, Victoria, Australia; 4 Howard Florey Institute, The University of Melbourne, Victoria, Australia; Harvard Medical School, United States of America

## Abstract

**Background:**

There is growing interest in the nature of slow variations of the blood oxygen level-dependent (BOLD) signal observed in functional MRI resting-state studies. In humans, these slow BOLD variations are thought to reflect an underlying or intrinsic form of brain functional connectivity in discrete neuroanatomical systems. While these ‘resting-state networks’ may be relatively enduring phenomena, other evidence suggest that dynamic changes in their functional connectivity may also emerge depending on the brain state of subjects during scanning.

**Methodology/Principal Findings:**

In this study, we examined healthy subjects (n = 24) with a mood induction paradigm during two continuous fMRI recordings to assess the effects of a change in self-generated mood state (neutral to sad) on the functional connectivity of these resting-state networks (n = 24). Using independent component analysis, we identified five networks that were common to both experimental states, each showing dominant signal fluctuations in the very low frequency domain (∼0.04 Hz). Between the two states, we observed apparent increases and decreases in the overall functional connectivity of these networks. Primary findings included increased connectivity strength of a paralimbic network involving the dorsal anterior cingulate and anterior insula cortices with subjects' increasing sadness and decreased functional connectivity of the ‘default mode network’.

**Conclusions/Significance:**

These findings support recent studies that suggest the functional connectivity of certain resting-state networks may, in part, reflect a dynamic image of the current brain state. In our study, this was linked to changes in subjective mood.

## Introduction

The success of functional magnetic resonance imaging (fMRI) as a non-invasive brain mapping technique has relied on its ability to detect local changes in evoked neural activity that are expressed indirectly in the blood oxygen level-dependent (BOLD) response [1]. In fMRI experiments, investigators typically set out to characterize regional BOLD signal changes to specific stimuli or tasks as ‘activations’ above a resting baseline or control state - an approach that has been influential in demonstrating how different brain systems respond to different types of external stimulation [2]. A recent departure from this approach has been to examine the so-called ‘resting-state’ dimension of fMRI and the appearance of slow synchronous variations of the BOLD signal (∼0.04 Hz) that occur prominently in the absence of external stimulation or tasks [3].

Current interest in these slow fluctuations in fMRI studies centers on the belief that they may represent some underlying or intrinsic form of brain functional connectivity in discrete neuroanatomical systems [4]–[7]. This was first suggested in fMRI studies of primary sensory cortices, where it was shown that these regions could be distinguished on the basis of their spontaneously correlated activity patterns under resting-state conditions [8]–[12]. These findings have since been extended to other higher cortical systems comprising putative language, attention and memory-related regions [6], [13]–[17] and have been recently identified in studies using independent component analysis (ICA); a data-driven technique that has shown value in isolating these networks from common artifacts in the fMRI time-series [18]–[22]. Taken together, these findings indicate that many of the brain systems that are routinely implicated in task-related fMRI experiments can also be defined on the basis of their ongoing spontaneous activities, or as distinct ‘resting-state networks’ (RSNs).

Although these RSNs appear to be relatively enduring phenomena [5], they may also demonstrate relevant changes in their functional connectivity depending on active changes in the brain state of subjects during scanning. This has been suggested in recent studies of steady-state and continuous task performance designs, where the functional connectivity of certain RSNs has been shown to increase or decrease in a task-consistent manner in response to changes in performance demands [13], [23]–[25] see also [26]. What is currently less known, but also likely as suggested by Waites and colleagues [26], is that relevant changes in the functional connectivity of RSNs may emerge due to changes to one's subjective experience during fMRI experiments, such as feeling states.

In the current study, we sought to test the influence of a change in subjective state, specifically mood state, on the functional connectivity of several RSNs as described in fMRI resting-state studies. To do so, we assessed healthy subjects during two continuous fMRI acquisitions while they participated in a modified version of the mood induction paradigm described by Damasio and colleagues [27]. With this task, subjects were instructed to perform two specific autobiographical memory recalls of a neutral (scan 1) and sad (scan 2) emotional event – the latter inducing significant and specific changes in their subjective mood state compared to the neutral condition. Because both tasks were acquired in a resting-like fMRI design, we were able to test for relevant changes in the functional connectivity of several RSNs as described in recent studies [18]–[22].

Based on existing findings from emotion-mood induction imaging studies, we made broad two predictions about the nature of functional changes that might be expected to emerge in association with sad mood induction. Firstly, we predicted that self-generated sadness would increase functional connectivity of a recently characterized RSN that consists largely of paralimbic regions, including the anterior insula and dorsal anterior cingulate cortices [6]. Co-activations of these two regions has been a consistent finding in existing emotion-mood induction experiments [28] and fits well with the idea that both structures represent major nodes of a cortical network for interoceptive awareness and feeling states [6], [29], [30]. Secondly, because the process of inducing changes in mood state via memory recall techniques requires explicit and intense cognitive effort [27], [31], [32], we expected that this may reduce the strength of functional connectivity of the ‘default mode network’. This RSN, which traditionally has been reported as a deactivation in task-related imaging studies, has also shown evidence for changes (i.e. decreases) in its global spontaneous activity pattern when subjects perform cognitively demanding tasks [25], [33]. To test these predictions and to extend our exploratory analysis based on ICA, we also performed specific cross-correlation analyses (CCAs) of functional connectivity among key regions of interest in these two RSNs.

## Results

### Behavioral

**Figure 1:**
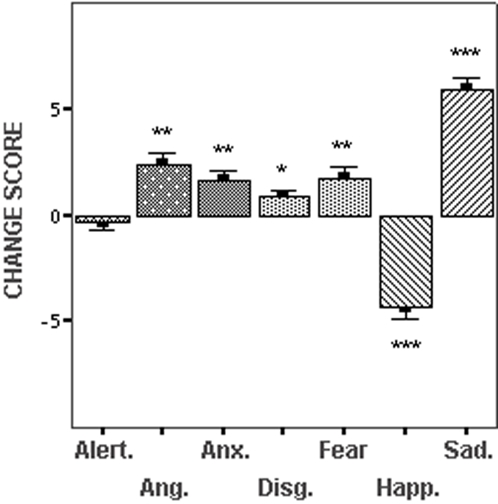
Subjective Mood State. Relative mean change scores (possible range±0–10) and the standard error of the mean for seven self-report dimensions that were used to quantify subjects' mood state after the neutral and sad recall conditions. The magnitude of statistical differences for each dimension (assessed by paired samples *t* tests) is referenced by * = p<0.01; ** = p<0.001; *** = p<0.0001.

Subjects' mood state was assessed directly after the neutral recall and sad recall conditions by verbal response to an 11-point rating scale of the seven dimensions, ‘alertness’, ‘anxiety’, ‘happiness’, ‘sadness’, ‘fear’, ‘anger’ and ‘disgust’. Scale range was from 0–10, with 0 reflecting ‘not at all’ and 10 reflecting ‘extremely’. The effect of task conditions on subjects' mood state was assessed in a two-way repeated-measures analysis of variance with mood dimensions and condition type (neutral and sad recall) as within-subject variables, which indicated a significant interaction (F(1, 6) = 41.21, p = 0.0001). Post-hoc t tests showed no significant change in subjective ratings of ‘alertness’ between the neutral and sad recall conditions [t (1, 23) = 0.67, p = 0.51], but significant changes in self-reported levels of anxiety [t (1, 23) = −3.78, p = 0.001]; happiness [t (1, 23) = 7.47, p = 0.0001], sadness [t (1, 23) = −11.44, p = 0.0001]; and fear [t (1, 23) = −3.55, p = 0.002]. In a pairwise comparison of the change scores for each mood state dimension, the magnitude of change for self-reported sadness between the neutral and sad recall conditions was found to be greater than for each of the other dimensions (p value range <0.023 to 0.0001). The direction and magnitude of self-report change scores between the neutral and sad recall conditions are provided in Figure 1.

### Functional MRI

fMRI-BOLD data were analyzed using combined group ICA and statistical parametric mapping techniques. Group ICA was performed for each fMRI experimental run separately (neutral and sad recall), generating two sets of group ICA results that were investigated further with statistical comparisons. To confirm our two study predictions and to compliment the exploratory ICA approach, specific pairwise CCAs for key regions of the ‘paralimbic’ and ‘default mode’ RSNs were performed. Full details of this analysis strategy are described in the Materials and Methods section.

### Spatial Identification and Assessment of Resting-State Networks

**Figure 2:**
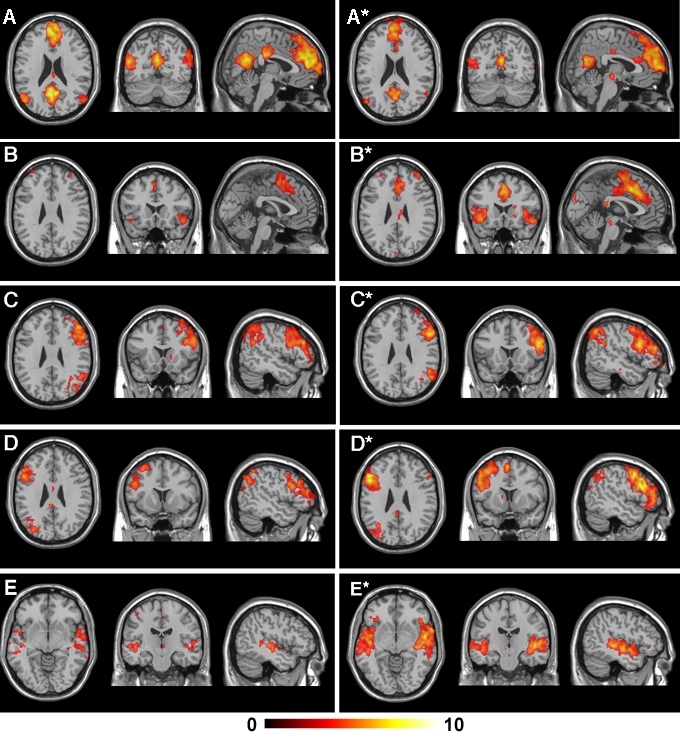
Common Resting-State Networks. Global functional connectivity maps of the common RSN patterns that were identified in the mood induction experiments (p _FDR_ *<*0.05). The left-most panel displays axial, coronal and sagittal views of the pattern under the neutral recall condition, while the right-most panel displays each corresponding pattern under the sad recall condition. All images are presented on a high-resolution single-subject MRI in standard neuroanatomical space (Montreal Neurological Institute, Colin-27). Corresponding color bars indicate the t score ranges of the displayed maps. Images are displayed in neurological convention (left = left).

From the group ICAs, we identified five significant independent component patterns of interest (i.e., RSNs) that were common to both of the task conditions (Figure 2). These five patterns reproduced the majority of cortical RSNs that have been described in recent studies applying this method to fMRI resting-state conditions [18]–[21], [34]. We also identified two further RSNs involving the primary visual and sensorimotor cortices, however these were found to be less reproducible between the two task conditions, in particular, during the sad recall experiment (see Figure S1).

Before testing for possible mood state-related changes in the functional connectivity pattern of the five common RSNs, we examined the relative stability of this measurement by performing a simple split-half analysis of both of the datasets, repeating the procedure of the original group ICAs. This analysis was considered necessary because although measurements of functional connectivity of RSNs have demonstrated spatial consistency across fMRI studies, it is less clear how reliable these measurements are in detecting changes of functional connectivity strength in the context of repeated measurements and task performance. To assess the reproducibility of our main effects using group ICA (Figure 2), we compared the findings of each split-half analysis qualitatively (by visual inspection) as demonstrated in Figure S2. For both datasets, we observed an 80% reproducibility of the five common RSN patterns and non-reproducibility in one of the split halves for two networks during the neutral and sad recall conditions. For the RSN patterns that reproduced in both split halves, there appeared to be good agreement (similarity) in the strength of functional connectivity estimated within-condition (neutral or sad recall), as well as apparent changes in the extent and magnitude of functional connectivity between these conditions as suggested from the original full-sample analysis.

**Figure 3:**
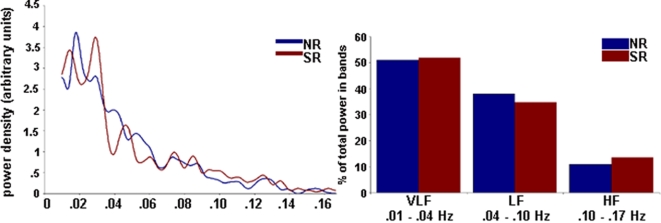
Temporal Analysis of Resting-State Networks. *Left*: A representative mean power spectral density plot for one component of interest (default mode network; Figure 2 A and A*). For all networks, the highest power density was observed below 0.04 Hz. *Right*: Proportion of power in the three non-overlapping frequency bands for the default mode RSN associated with the neutral and sad recall conditions. All identified components showed the highest percentage of power in the very low frequency band. LF = low frequency; VLF = very low frequency; HF = higher frequency. NR = neutral recall; SR = sad recall.

The relative stability of the common RSN fluctuations was also assessed in the temporal domain through a detailed analysis of the power spectral distribution (PSD) of each network's associated activity time-course after detrending. For each subject, the predominant power-spectral distribution of each RSN time-course was estimated as corresponding to the ‘very low frequency’ (0.01–0.04 Hz), ‘low frequency’ (>0.04–0.1 Hz) and ‘higher frequency’ (>0.1–0.17 Hz) domains. All networks had predominant power density distributions in the very low frequency domain, similar to recent fMRI studies of spontaneous resting-state conditions [8], [9], [13], [18]–[20], [35], [36]. An example of this assessment is provided in Figure 3. For the five common RSN pairs, there were no significant differences in their relative proportions of power distributed within this range (p value range = 0.19–0.58), indicating that these signal variations were similar between the neutral and sad recall conditions.

**Figure 4:**
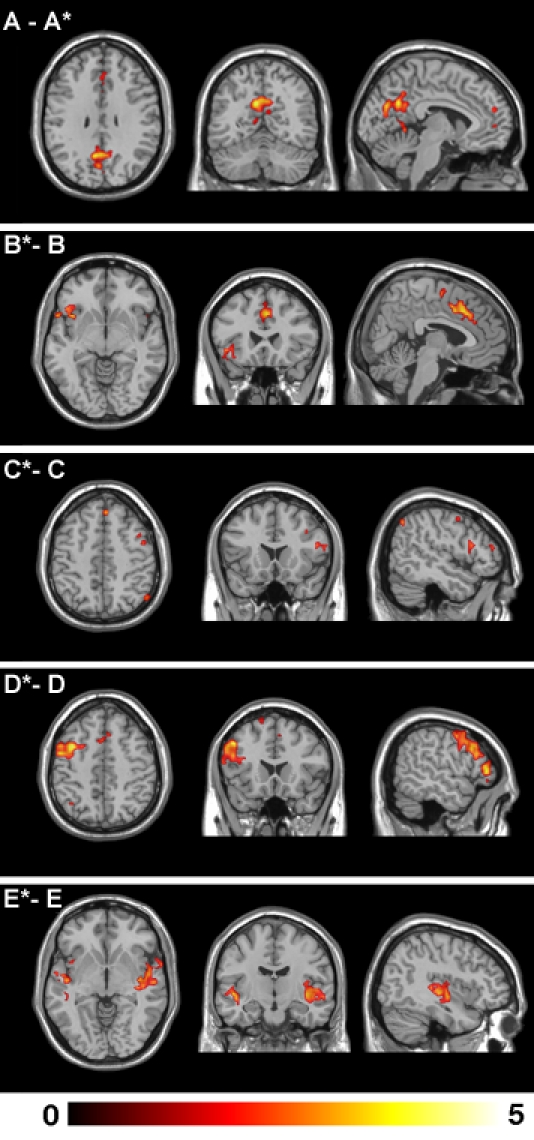
Modulation of Resting-State Networks by Mood Induction. Global functional connectivity differences observed between the neutral and sad recall conditions for the common RSNs. The top panel A - A* indicates an apparent *decrease* in functional connectivity of the default mode network between the neutral and sad recall conditions. All other panels indicate apparent *increases* of functional connectivity during the sad compared to neutral recall condition. All images are presented on a high-resolution single-subject MRI in standard neuroanatomical space (Montreal Neurological Institute, Colin-27). Corresponding color bars indicate the t score ranges of the displayed maps. Images are displayed in neurological convention (left = left).

Figure 4 shows the relative changes of functional connectivity that were observed in each RSN between the two task conditions (p<0.005). These changes occurred as either increases or decreases in the statistical magnitude and/or extent of regional clusters showing correlated activities in the very low frequency domain (<0.04 Hz). The anatomical co-ordinates of all regional RSN clusters, including their z score magnitudes and extents are reported in Table 1. These five common networks can be summarized as follows.

**Table 1:** Regional Statistical Mapping of Resting-State Networks

Neutral Recall	Anatomy			Stats¶		Sad Recall	Anatomy			Stats¶		Difference	Anatomy			Stats∥	
**A**	**X**	**Y**	**Z**	**CS**	**Z**	A*	**X**	**Y**	**Z**	**CS**	**Z**	A - A *	**X**	**Y**	**Z**	**CS**	**Z**
Posterior cingulate gyrus (31)	−9	−63	27	702	6.97	Medial frontal gyrus (10)	−3	66	6	1841	6.58	Posterior cingulate gyrus (31)	−9	−63	27	226	4.1
Medial frontal gyrus (10)	−6	51	21	1929	6.83	Posterior cingulate gyrus (31)	0	−69	18	325	6.19	Caudate nucleus	9	12	3	26	3.61
Angular gyrus (39)	48	−78	30	291	6.07	Angular gyrus (39)	−51	−78	33	128	5.47	Angular gurus (39)	45	−78	36	61	3.53
Posterior cingulate gyrus (23)	0	−27	30	196	6.04	Brainstem	−3	−21	−9	84	4.7	Medial frontal gyrus (10)	−3	48	21	251	3.34
Angular gyrus (39)	−51	−75	21	292	5.86	Superior temporal gyrus (22)	30	27	−24	34	4.46	Posterior cingulate gyrus (2)	0	−30	30	41	3.22
Caudate nucleus	12	12	6	312	5.31	Mid cingulate cortex (23)	3	−21	36	40	4.37	Caudate	−12	9	6	18	3.05
Superior temporal gyrus (22)	−39	24	−30	126	4.81	Caudate	12	12	9	9	3.98	Precuneus (31/7)	6	−66	18	11	2.93
Superior temporal gyrus (22)	30	21	−30	44	4.6	Angular gyrus (39)	−45	21	−24	10	3.98	Angular gyrus (39)	−54	−63	21	62	2.93
Superior frontal gyrus (8)	42	15	42	42	4.48	Superior frontal gyrus (8)	21	24	60	8	3.74						
Middle frontal gyrus (9)	−24	45	36	33	4.38	Superior temporal gyrus (28)	−36	18	−24	8	3.65						
Superior frontal gyrus (6)	−45	9	54	9	3.8												
Thalamus	6	−18	9	10	3.55												
**B**						B*						B* - B					
Superior frontal gyrus (6)	3	3	51	81	6.05	Dorsal anterior cingulate (32)	3	6	42	879	7.27	Anterior insula cortex	−42	15	−9	50	3.64
Anterior insula cortex	45	15	−12	37	4.77	Anterior insula cortex	−42	15	−9	436	6.08	Anterior insula/operculum	−57	12	−3	22	3.47
Dorsal anterior cingulate (32)	−6	9	45	10	4.74	Anterior insula cortex	39	24	0	258	5.07	Dorsal anterior cingulate (32)	3	−6	42	168	3.44
Media frontal gyrus (9)	−36	51	24	9	4.69	Brainstem	3	−30	−15	27	4.91	Superior frontal gyrus (6)	3	−9	60	21	2.97
						Parastriate cortex (19)	−3	−93	30	16	4.71						
						Caudate	18	15	12	14	4.61						
						Medial frontal gyrus (10)	24	51	18	9	4.55						
						Medial frontal gyrus (9)	36	45	27	69	4.49						
						Putamen	18	15	−3	22	4.41						
						Medial frontal gyrus (9/10)	−30	54	24	24	4.05						
**C**						C*						C*-C					
Medial frontal gyrus (9)	48	30	27	928	5.77	Medial frontal gyrus (8)	48	12	51	1295	6.27	Superior frontal gyrus (8)	3	36	51	53	3.04
Inferior parietal cortex (40)	54	−45	54	511	5.08	Inferior parietal cortex (40)	51	−66	45	738	6.21	Inferior parietal cortex (40)	51	−66	45	36	2.8
Thalamus	6	−6	15	30	4.7	Superior frontal gyrus (8)	3	33	48	265	5.72	Inferior frontal gyrus (44/45)	57	18	15	30	2.6
Medial frontal gyrus (10)	36	57	15	9	4.64	Anterior insula cortex	39	24	−9	59	5.02	Anterior insula cortex	39	24	−9	11	2.54
Caudate	12	6	9	14	4.59	Thalamus	−9	−3	12	17	4.61						
Parastriate cortex (19)	42	−75	27	28	4.41	Thalamus	3	−6	12	23	4.43						
Superior frontal gyrus (8)	6	24	42	51	4.38	Superior temporal gyrus (22)	63	−42	9	9	4.07						
Thalamus	−6	−6	15	13	4.34	Medial frontal gyrus (9/10)	27	57	27	15	3.82						
Anterior insula cortex	36	24	−3	14	4.27												
Inferior frontal gyrus (11)	45	42	−18	11	4.04												
Superior frontal gyrus (8)	12	36	48	10	3.96												
**D**						D*						D*-D					
Inferior parietal cortex (39/40)	−48	−72	42	304	5.39	Inferior frontal gyrus (44/45)	−54	24	27	1291	6.59	Medial frontal gyrus (6/8)	−39	3	45	849	3.92
Medial frontal gyrus (9)	−48	24	30	325	5.33	Superior frontal gyrus (6)	−6	9	60	221	5.65	Superior frontal gyrus (8/9)	−3	6	57	98	3.26
Medial frontal gyrus (6/8)	−27	9	63	188	5.17	Inferior parietal cortex (39)	−42	−72	45	284	5.1	Medial frontal gyrus (9)	−48	45	21	15	2.83
Posterior cingulate cortex (23)	−6	−39	27	19	4.73	Posterior cingulate gyrus (23)	−3	−45	27	10	4.25	Inferior parietal cortex (7)	−33	−90	36	28	2.78
Medial frontal gyrus (9)	−21	51	39	36	4.4	Putamen/globus pallidus	−18	0	6	6	4.22	Inferior parietal cortex (40)	−42	−54	39	8	2.69
						Caudate	−15	9	6	7	4.2	Medial frontal gyrus (9)	−24	15	69	18	2.59
						Inferior frontal gyrus (47)	−30	27	−6	12	4.11						
						Superior frontal gyrus (8)	−18	18	66	8	3.84						
						Medial frontal gyrus (6)	−27	6	42	5	3.78						
						Medial frontal gyrus (8)	−18	36	48	6	3.78						
**E**						E*						E*-E					
Superior temporal gyrus (42)	−60	−33	0	31	5.68	Medial temporal gyrus (21)	−51	−6	−6	1125	6.27	Posterior insula cortex	39	−12	6	287	3.79
Transverse temporal gyrus (41/42)	−48	−30	0	16	4.99	Superior temporal gyrus (42)	51	−36	0	806	5.8	Medial temporal gyrus (42)	−54	−9	0	79	3.63
						Posterior insula cortex	36	24	−6	19	4.48	Medial temporal gyrus (42)	−48	−36	3	45	2.65
						Hippocampus/amygdala	30	−9	−15	8	3.8						
F						G*						-	-	-	-	-	-
Parastriate cortex (18)	−9	−78	−9	315	5.38	Post-central gyrus (4/2)	3	−30	48	380	5.29	-	-	-	-	-	-
Parastriate cortex (18)	−18	−54	−27	10	4.33	Post-central gyrus (2)	−21	−39	75	45	4.39	-	-	-	-	-	-
Striate cortex (17)	9	−84	6	15	4.22	Post-central gyrus (1/2)	54	−27	60	160	4.28	-	-	-	-	-	-
Striate cortex (17)	15	−93	9	14	4.21	Superior frontal gyrus (6)	57	0	39	26	4.18	-	-	-	-	-	-
						Pre-pariatal cortex (5)	−36	−39	66	90	4.09	-	-	-	-	-	-
						Thalamus	3	−3	6	23	4	-	-	-	-	-	-
						Pre-central gyrus (4)	51	−12	57	12	3.97	-	-	-	-	-	-
						Post-central gyrus (2)	−18	−30	75	8	3.78	-	-	-	-	-	-
						Superior frontal gyrus (6)	30	−15	66	11	3.64	-	-	-	-	-	-
						Superior parietal cortex (7)	21	−75	57	8	3.55	-	-	-	-	-	-
						Pre-parietal cortex (5/7)	9	−60	66	9	3.44	-	-	-	-	-	-

** Activation co-ordinates (x, y, z) are given in MNI space (Montreal Neurological Institute). Numbers given in parentheses reflect approximate Brodmann Area (BA) locations.

¶ Magnitude and extent statistics correspond to a minimum p **_FDR_** *<*0.05 (range 0.05 to 0.0001);

∥ Magnitude and extent statistics correspond to a minimum p<0.005 (range −0.005 to 0.0001).

**Default Mode Network**: included primary clusters in the posterior cingulate and medial prefrontal cortices as well as the angular gyri. Specific regions that showed decreased functional connectivity during the sad recall condition (A - A*) included the dorsal and ventral posterior cingulate cortex, bilateral angular gyri, ventral medial frontal cortex, caudate nucleus and putamen.

**Paralimbic Network:** included clusters located primarily in the dorsal anterior cingulate and insula cortices, supplementary motor area and dorsal medial frontal cortex. Specific regions that showed increased functional connectivity during the sad recall condition (B* - B) included the dorsal anterior cingulate and supplementary motor area, and left anterior insula and opercular region.

**Right Frontoparietal Network:** included clusters lateralized to the right hemisphere, including the lateral prefrontal (medial and inferior frontal gyri), the inferior parietal cortices, caudate nucleus and supplementary motor area. Specific regions that showed increased functional connectivity during the sad recall condition (C* - C) included the right inferior, medial and superior frontal gyrus and inferior parietal cortex.

**Left Frontoparietal Network:** included clusters lateralized to the left hemisphere, including the lateral prefrontal cortex (superior, medial and inferior frontal gyri), inferior and superior parietal cortices, thalamus and caudate nucleus. Specific regions that showed increased functional connectivity during the sad recall condition (D* - D) included the left medial and superior frontal gyrus and inferior parietal cortex.

**Auditory Cortex Network:** included clusters located primarily in the lateral superior temporal cortex and posterior insular cortex. Specific regions that showed increased functional connectivity during the sad recall condition (E* - E) included the right posterior insula and left mid-temporal cortex.

### Confirmatory Cross-Correlation Analysis

To further test our predictions of changes in functional connectivity due to sad mood induction and to confirm our exploratory ICA findings, we performed seed-based CCAs between major regions of interest (ROIs) in the ‘paralimbic’ and ‘default mode’ networks (see Materials & Methods). Briefly, for the ‘paralimbic’ RSN, this involved extracting an average reference time-course from our primary seed ROI located in the dorsal anterior cingulate cortex and assessing for differences in its cross-correlation strength between the two task conditions with a target ROI in the right anterior insula cortex. For the ‘default mode’ RSN, the same procedure was repeated but with the seed ROI located in the posterior cingulate cortex and target ROI in the medial frontal cortex. All ROI placements and dimensions were determined via a conjunction analysis of the exploratory ICA connectivity maps, which identified those regions whose activity was highly and jointly significant to both conditions (p **_FDR_** *<*0.05).

**Figure 5:**
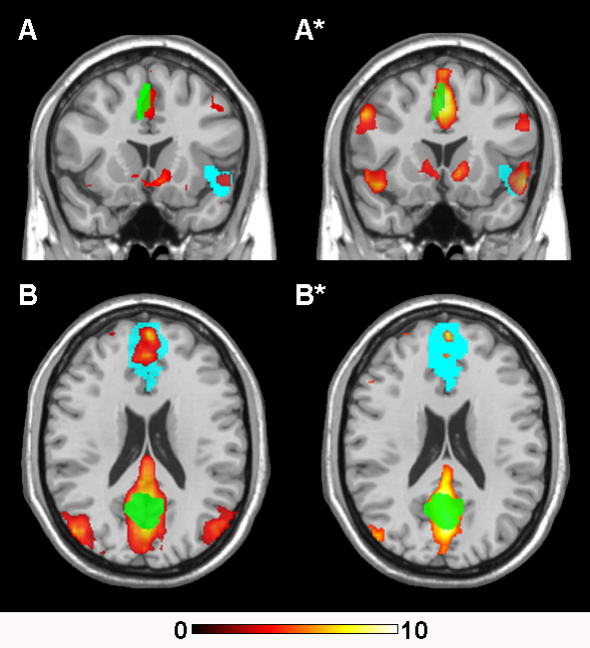
Cross-Correlation Analysis of Specific Resting-State Networks. Regional functional connectivity maps of the ‘paralimbic’ (A) and ‘default mode’ (B) RSNs. Green clusters represent both of the seed ROIs while the blue clusters represent their respective target ROIs. Both ROI types are overlaid on the regional functional connectivity maps derived from the cross-correlation analyses. All images are presented on a high-resolution single-subject MRI in standard neuroanatomical space (Montreal Neurological Institute, Colin-27). Corresponding color bars indicate the t score ranges of the displayed maps. Images are displayed in neurological convention (left = left).

Consistent with the group ICA findings, both RSN patterns were reproduced by the seed-based cross-correlation approach (Figure 5). For the paralimbic network, we observed a significant increase of functional connectivity between the dorsal anterior cingulate and right anterior insula cortex in the sad recall relative to neutral recall condition (x, y, z = 48, 21, −15; z = 2.86; 24 voxels). By comparison, for the default mode network, there was also decreased of functional connectivity between the posterior cingulate and medial frontal cortex in the sad relative to neutral recall condition (x, y, z = 6, 54, 12; z = 2.93; 23 voxels).

## Discussion

There is growing interest in spontaneous fluctuations of the fMRI-BOLD signal and its potential to encode an intrinsic form of brain functional connectivity in discrete brain systems. While efforts so far have focused on clarifying the physiological basis and spatial replicability of these signals across studies [8], [9], [16]–[20], it is less clear how changes or differences in the behavioral state of subjects during scanning may be represented in these so-called ‘resting-state networks’ (RSNs). Such data will ultimately be important for interpreting the functional meaning of spontaneous activity fluctuations in basic and clinical fMRI studies.

The current findings add to existing work by demonstrating that measurable changes in the functional connectivity of certain RSNs emerged in healthy subjects undergoing an experimental manipulation of their subjective (i.e., mood) state during resting-like fMRI conditions [13], [23]–[26]. In each of the five common RSNs identified, we observed significant changes in their global functional connectivity pattern between the neutral and sad mood induction conditions, appearing either as regional increases or decreases of the magnitude and extent of correlated voxels in the very low frequency domain (<0.04 Hz). Changes of this nature are in good agreement with other studies to have compared fMRI steady-state and continuous task performance conditions against rest [13], [23]–[26], but whereas in the current study, we describe these changes in the context of a multivariate analysis that has characterized changes in several networks simultaneously.

The finding of increased functional connectivity of the ‘paralimbic’ RSN during the subjective experience of sadness fits remarkably well with existing imaging studies of mood-emotion induction tasks [28], and current ideas of the functional relevance of this brain system in humans [6], [29], [30]. This RSN consists primarily of paralimbic structures including the dorsal anterior cingulate cortex and anterior insula-operculum; regions that consistently co-activate in functional imaging studies in response to personally salient stimuli and various forms of cognitive and affective engagement 29, 30, 37, 38. The frequency and commonality of their co-activation in imaging studies has been taken to support idea that both regions represent major nodes of a cortical network for interoceptive awareness; a network thought to mediate subjective feeling states arising from brain representations of bodily responses [6]. In keeping with this, the finding of increased functional connectivity of these regions in the current study during the sad memory recall may represent a general change in the subjective state of participants during this condition, rather than a specific correlate of increased sadness *per se*. This would be consistent with other mood induction imaging studies, where the co-activation of these regions has also been linked to other factors, such as the degree of mental effort and cognitive demand that is evoked by different paradigms, in particular those based on emotional memory recall/recollection [28].

Also supporting the initial predictions of our study was the observation of reduced functional connectivity in midline regions of the ‘default mode network’ when comparing the sad recall and neutral recall conditions. This pattern appears to be consistent with the common finding of task-induced deactivations of this network that have been reported in many other functional imaging studies [39]–[44]. Deactivations of this network emerge routinely in task-related imaging studies when periods of rest or passive imaging states are compared with periods of cognitively demanding tasks, implying greater functional engagement of these regions during the passive imaging states [41]. This interpretation may carry over the current experiment, with the neutral recall condition representing a more passive or less cognitively demanding task than the sad memory recall, as described in other studies [27], [31], [32]. That such a ‘cognitive’ aspect to the sad memory recall condition may have reduced functional connectivity of the default mode network is also consistent with two recent studies that have compared the functional connectivity of this network during resting-state and working memory task conditions using equivalent methods (i.e., ICA [25], [33]). However, the extent to which our findings also mark a deviation from the resting-state activity of this network was not directly tested, and thus it is possible that both task conditions may have reduced its resting functional connectivity, albeit to different extents.

In addition to the observed changes in the ‘paralimbic’ and ‘default mode’ RSNs, functional connectivity increases were also identified in association with the sad recall conditions in three other previously described RSNs, including left and right frontoparietal ‘dorsal attention’ systems and a bilateral ‘auditory cortex’ network [15], [18]–[20]. Like the aforementioned networks, there is also intuitive appeal in the nature of these findings, where for instance the greater apparent increase of functional connectivity in the left versus right frontoparietal network during sad memory recall agrees with the pattern of lateralized cortical activations that have been reported in existing imaging studies of autobiographical and emotional memory recall [45]. Similarly, for the auditory cortex RSN, functional connectivity changes were mapped primarily to the mid and posterior insula cortex, which is consistent with other evidence linking this region, rather than primary auditory cortex, to the experience of emotional intensity during mental imagery [46]. Nevertheless, despite the plausibility of these findings, they should also be considered preliminary at this point. This is said for two reasons: Firstly, compared to the paralimbic and default mode networks, the identification of these other RSNs demonstrated a tendency towards lower reproducibility (80%) in our simple split-half analyses shown in Figure S2. Secondly, and importantly, we report these findings in the context of a novel and exploratory imaging analysis (group ICA) with no formal hypotheses regarding the influence of our experiment on the activity of these networks. Therefore, we suggest that these findings are in need of replication.

Extending the above discussion, we have also described variable effects of the mood induction experiment on two frequently reported sensory RSNs involving visual and sensorimotor cortices (Figure S1). From our original group analyses (n = 24), both of these networks showed lower reproducibility than other RSNs to the extent they were not detected as a reliable signal source in association with the sad memory recall condition. Although others have commented on the variability of certain RSN measurements [26], the precise interpretation of this result in our study is not straightforward. For instance, it is possible that the lower reproducibility of these two networks reflects; *(i)* greater variability among subjects due to a true influence of task on their functional organization; *(ii)* greater variability in the measurement of their activities because of the specific analysis approach; or *(iii)* some interaction of both factors. The last two possibilities may also be considered more probable with the use of ICA algorithms compared to other functional connectivity methods (i.e. CCAs), given their greater analytic complexity (however, see [47]). We explored this by performing additional CCAs and split-half analyses for both of these sensory RSNs and observed similar pattern of low reproducibility for the sensorimotor network (Figure S3). Clearly, further and more sophisticated studies are needed to address the issue of reproducibility of different functional connectivity methods in fMRI studies and whether such approaches can be meaningfully integrated to provide a consensus imaging approach.

While evidence is accruing in support of a definite neuronal basis to the spontaneous signal fluctuations observed in fMRI experiments (for a discussion see [3]), the influence of non-neuronal factors on the measurement of these signals should not be ruled out. That is, although methods such as ICA have shown good utility in separating potential noise sources in resting-state fMRI studies, including aliased physiological signals associated with the cardiac (vascular pulsation) and respiratory (chest movement) cycles [18], [48], we cannot exclude an influence of these signals in the our study without direct physiological monitoring. We were also unable to quantify the influence of slow variations in subjects' breathing rate and volume (reflected as small variations in end-tidal arterial CO_2_) on the extracted BOLD signal measurements [49]. While recent work suggests that the effect of aliased physiological signals on the estimation of low frequency BOLD signal fluctuations may be small [48], it will be important for future studies to account for these effects directly with physiological monitoring.

Although much remains to be known about these slow BOLD signal fluctuations, they are becoming increasingly seen as a major source of non-modeled variance in fMRI studies that may provide some new insight into the functional connectivity or organization of discrete neuroanatomical systems [4], [5]. Our findings support the proposal of recent fMRI studies which also indicate that the functional connectivity of these resting-state networks may, in part, represent a dynamic image of the current brain state [26]. Considering the growing interest of resting-state (or resting-like) fMRI protocols to compare brain functional connectivity estimates in healthy and clinical populations [50]–[54], further work investigating the state and trait behavioral correlates of these activity fluctuations is necessary.

## Materials and Methods

### Subjects

Twenty-four, right-handed, healthy volunteers were recruited for this study (12 female; mean age and SD = 31.0±8.3 years). All subjects spoke English as a first language and had no history of neurological disorder or psychiatric illness. Subjects' mean education level and general intelligence scores [55] were 15.3±2.4 yrs and 114±10.8, respectively. All subjects gave written, informed consent to participate in the study, which was approved by the Mental Health Research Institute of Victoria and Melbourne Health Research and Ethics Committees.

### Mood Induction Paradigm

We used a modified version of the paradigm reported by Damasio and colleagues [27]. During an initial interview session, one week prior to scanning, participants were told that they would be required to think about two events in their past - one especially non-emotional experience and one especially sad experience. Subjects were told that they would be asked to provide broad (not detailed) accounts of the contents of their imagery after scanning. As described in Damasio et al., there was no attempt to constrain the themes artificially by limiting the recall to episodes involving the same persons or places or a certain time span, because we were interested in gaining access to the autobiographical episodes that the subjects considered to be emotionally most powerful.

For the neutral recall condition, subjects were asked to recall in detail a specific but unemotional day in their lives (e.g., a typical day at work in which everything is routine) assisted by the musical piece ‘Chopin Waltzes numbers 11 and 12’ (played at half-speed). Subjects were asked to recall this day chronologically, i.e., from waking up in the morning, to preparing breakfast, getting dressed, hearing the news, leaving for work, arriving at work etc. For the sad recall condition, subjects were asked to recall in detail a specific, personal episode or event of particular sadness in their lives and to attempt to re-experience the emotions, aided by the musical piece ‘Russia under the Mongolian Yoke’ by Prokofiev. To encourage that true autobiographical recalls were generated for both conditions, subjects were instructed not to imagine or interject untrue events (i.e. worst/best case scenarios) but rather to think about events that actually happened.

During scanning, subjects were instructed to close their eyes and attempt to recall and re-experience the specific neutral or sad emotional episodes (i.e., scan 1 = neutral recall, scan 2 = sad recall). Subjects were told to actively visualise, think and ruminate about the specific episodes rather than to concentrate on their feelings of relaxedness or sadness. They were also instructed to maintain as long as they could these specific feeling states until the end of the scanning period, i.e., after they had indicated each scan to commence (see further). Each condition commenced with the selected music pieces being played. The choice of music to accompany the sad recall condition was based on previous studies indicating that this piece, together with the sad memory recall, consistently and specifically induced dysphoric mood without co-producing other related emotional states (e.g. anxiety) [56]–[58]. This approach to mood induction, including the specific sad music piece, have been used successfully in previous PET and fMRI studies (e.g., [59], [60]), albeit to assess the influence of induced mood states on latter cognitive task performance. In our study, the presentation of music pieces during the pre-scanning (induction) period was also employed to reduce distraction to surrounding noise in the MRI environment.

Each musical piece (presented via headphones) was played for between one and five minutes on average before each of the four-minute scans commenced, depending on the time it took for the participant to achieve the mood state. Scanning commenced only after the subject indicated (via button press) that they felt a certain intensity of neutral or sad mood had been reached. While we allowed up to five minutes for subjects to achieve the desired mood states, in practice, all subjects indicated to commence the scans within a three-minute period. For the neutral recall condition, this period lasted on average between one to two minutes while for the sad recall condition this lasted typically two to three minutes. Throughout the scanning sequences, subjects lay in a relaxed position and were instructed to keep their eyes closed at all times without falling asleep. Mood state ratings (VAS) and alertness was assessed by self-report and communication with the subjects between the neutral and sad recall conditions. All subjects spent approximately 30 minutes in a mock scanner prior to the actual study period in order to familiarize themselves with the MRI environment.

### Behavioral Measures

Subjects' mood state was assessed by verbal response to an 11-point rating scale of the seven dimensions, ‘alertness’, ‘anxiety’, ‘happiness’, ‘sadness’, ‘fear’, ‘anger’ and ‘disgust’. Scale range was from 0–10, with 0 reflecting ‘not at all’ and 10 reflecting ‘extremely’. In a debriefing session following scanning, subjects were also asked to indicate the extent to which they were actively engaged in the sad autobiographical recall, rating on an 11-point scale (0–10) their relative ‘ease of inducing sadness’ (mean±SD = 7.1±2.1); ‘similarity of feelings when compared to actual life event/episode’ (mean±SD = 6.7±2.1); and the ‘approximate proportion of time sadness was maintained throughout the four-minutes’ (mean±SD = 5.5±2.1). Behavioral data were analyzed using the Statistical Package for the Social Sciences v. 11 (SPSS, Chicago, Illinois).

### Imaging Acquisition

Individual MRI sequences were acquired in a single scanning session using a 3 Tesla GE Signa Horizon LX whole body scanner (General Electric, Milwaukee, WI, USA). Subjects' heads were fixed using a Velcro® strap over the forehead. Functional MRI data were acquired as a series of single shot gradient-recalled echo planar imaging volumes providing T2*-weighted BOLD contrast (repetition time, 3,000 ms; echo time, 40 ms; flip-angle 60°; field of view, 24 cm; voxel size, 1.875×1.875×4.0 mm; number of slices, 25). For each condition, the functional time-series consisted of eighty consecutive whole-brain images (4-minutes duration) after automatically discarding the first four images in each run to allow the magnetization to reach equilibrium. Data were transferred to an IBM-PC workstation for image pre-processing and analysis.

### Preprocessing & Analysis

Image pre-processing was performed in SPM5 (www.fil.ion.ucl.ac.uk/spm/software/spm5/), and involved motion correction, spatial normalization and smoothing using a Gaussian filter (full-width, half-maximum, 5 mm). Motion correction was performed by aligning (within-subject) each time-series to the first image volume using a least squares minimization and a 6 parameter (rigid body) spatial transformation. We further compared the translation and rotation estimates (x, y, z) from both scans (neutral and sad recall conditions) using repeated measures analysis of variance (ANOVAs) to ensure equivalent data quality. Translation and rotation estimates (x, y, z) were all less than 1 mm or 1°, respectively. For translation estimates, we observed no main effect of condition (F(1, 23) = 1.43, p = 0.23) or translation estimate by condition interaction (F(1, 23) = 0.30, p = 0.74). For rotation estimates, we observed no main effect of condition (F(1, 23) = 3.59, p = 0.07) or rotation estimate by session interaction (F(1, 23) = 0.35, p = 0.71). Data were normalized to the standard SPM-EPI template and resliced to 3 mm isotropic resolution in Montreal Neurological Institute (MNI) space.

### Group ICA

Independent component analysis (ICA) is a data-driven statistical analysis method that is able to decompose high-dimensionality data, such as the fMRI time-series, into discrete signal and noise covariance components [61], [62]. Group ICA methods have gained recent attention in the analysis of fMRI studies because of their robust and flexible modeling nature, which has included several recent studies using ICA to specifically and systematically identify cortical RSN activity during fMRI resting-state conditions [18]–[21], [25], [34]. In addition to providing a data-driven and multivariate characterization of fMRI studies, ICA results also provide a measure of functional connectivity in such studies - ‘the temporal correlation between remote of neurophysiological measurements’ [63]. In the case of ICA, ‘functionally connected’ voxels or regions are defined spatially by their same dependency of temporal variation as a system, which is estimated in the higher-order (or complete) statistical sense (for a detailed discussion see [64]).

In this study ICA was performed using the Group ICA for fMRI Toolbox (GIFT v1.3b; http://icatb.sourceforge.net) run on Matlab 7, using methods and algorithms described in recent studies [65]–[67]. Briefly, a single ICA analysis was performed at the group level for the neutral and sad recall conditions separately, after subject-wise data concatenations, and back reconstruction of single-subject time courses and spatial maps from the raw data matrix [65]. As detailed in other recent studies [50], [65], [68], GIFT performs this procedure as three distinct stages: *(1)* data reduction, *(2)* application of the ICA algorithm, and *(3)* back reconstruction.

During *Stage 1*, principal component analysis (PCA; with 3 reduction steps) was used to reduce individual subjects' data in dimensionality (for computational feasibility). The dimensionality of the data (number of components) was estimated using the minimum description length criteria tool incorporated in the GIFT package, which attempts to minimize mutual information between components (for details see [69]. For the neutral recall condition, data from each subject (n = 24) were firstly reduced from 80 to 21 dimensions, followed by a second concatenation into six groups of n = 4 subjects each. The dimensionality of each subgroup (i.e., n = 4 sets of 21 dimensions) was reduced from 84 to 21 dimensions using PCA. This was followed by an ultimate concatenation and reduction into one group with 21 components. For the sad recall condition, data from each subject (n = 24) were first reduced from 80 to 24 dimensions followed by a second concatenation into six groups (of n = 4 subjects), each of which was reduced from 96 dimensions to 24. This was followed by an ultimate concatenation and reduction to one group with 24 components.

In *Stage 2*, the estimation of independent sources was performed using the Infomax algorithm [70]. During this stage the spatially independent component maps were created, while during *Stage 3* of back reconstruction, individual subject image maps and time courses were estimated using the group solution to accurately represent the subject-to-subject variability existing in the data [65]. The resulting single-subject time course amplitudes were then calibrated (scaled) using the raw data to reflect percent fMRI signal strength, followed by normalization to z score values. In this process, the estimated time course is treated as the model and is fitted to the raw data using an intercept term. This fit is then used to scale (or normalize) the component images into z score units also reflecting the data's deviation from the mean, thus, enabling second-level random effects analyses to be performed.

### Spatial Identification of Resting-State Networks

For the neutral and sad recall conditions, ICA produced 21 and 24 maximally independent patterns (ICs) of spatiotemporally covariant BOLD signal changes, respectively. Both the spatial pattern and frequency spectra (see below) of each component were visually inspected to determine their appearance as potential RSNs or possible image artifacts. Noise ICs, which corresponded to distinct and clear image artifacts, were related to subject head motion and related susceptibility artifact at the frontal sinus; eyeball movement, cardiac-induced pulsatile fluctuation at the base of the brain and/or surrounding major vessels, and CSF signal fluctuation due the respiratory and cardiac cycles (see examples in [9], [18], [20], [48], [71]). For the neutral and sad recall conditions, 7 and 5 hypothesized RSNs were identified for further analysis, respectively.

### Significance Testing of Resting-State Networks

SPM5 was used to estimate second-level group RSN maps to compare the common RSNs for differences in their relative functional connectivity strength between the neutral and sad recall conditions. Group statistical maps were estimated for each RSN separately, by first entering each subject's respective independent component images (z score maps) into voxel-wise one-sample *t* tests (p **_FDR_** *<*0.05, corrected). To assess for differences in the functional connectivity pattern of common RSNs, a global mask was created by combining all regions from both RSN patterns that survived the previous correction of p **_FDR_** *<*0.05 [50]. Paired samples *t* tests were then used to test for differences in functional connectivity strength of the common RSNs restricted only to voxels contained in each global mask. Regional differences were considered significant if surviving p<0.005 (uncorrected), with a minimum cluster extent of at least 5 contiguous voxels.

### Temporal Analysis of Resting-State Networks

Initially, detrending was performed to remove signal drifts that occur below 0.01 Hz. As discussed in recent studies [19], transformation of group ICA associated time courses into the frequency domain is necessary because phase synchrony of these slow signal fluctuations cannot be assumed across subjects under resting-state conditions. By characterizing ICA associated time-courses on the basis of PSDs, inter-subject averaging becomes possible and, in turn, facilitates the assessment of frequency characteristics and differences between components. This served two basic purposes in the current study; (i) to confirm that all selected RSNs showed dominant PSDs in the expected ‘very low frequency’ domain [9], [36], [72], and (ii) to assess for any significant differences in the PSD parameters for the common RSN patterns identified in association with the neutral and sad recall conditions (i.e., which may reflect different sources of signal variation).

To perform this analysis, we derived the PSD estimate using a modified periodogram method (with a Hamming window, 240 s) for each subject's respective RSN time-series (i.e., 5×2 common RSN patterns+2 specific RSNs per subject = 12 in total). Components were normalized in energy (σ^2^ = 1) prior to the analysis. We also computed each RSN's average PSD across subjects, which showed good fitting to the a+bf^−1^ model previously described for raw BOLD signal analysis [73] with an *r* ^2^ range of 0.97 to 0.55. Dominant oscillating frequencies were observed as one or more clear peaks above this baseline, typically around 0.03 Hz. Our specific interest was to compare the proportion of power in this frequency domain among the common RSNs. To perform this comparison without an operator-driven bias, we divided our frequency range into three domains based on the literature; ‘very low frequency’ ranging between 0.01–0.04 Hz; ‘low frequency’ ranging between 0.04–0.10 Hz; and ‘higher frequencies’ ranging between 0.1–0.17 Hz [9], [36], [72]. For each extracted RSN, the proportion of the total power in each of these bands was estimated (see e.g., in Figure 3). Focusing on the ‘very low frequency’ range of interest, paired-samples Wilcoxon Signed Ranks Tests were used to assess for differences in the proportion of power across this frequency range between common RSNs.

### Confirmatory Cross-Correlation Analysis

Seed-based cross-correlation analyses (CCAs) were performed to assess for specific changes in functional connectivity strength among key ROIs (seed and target) in the ‘paralimbic’ and ‘default mode’ RSNs. The ROI placements and size (i.e., volume) were derived from a conjunction analysis in SPM5 of the ICA functional connectivity maps for each RSN, as described above. This analysis identified clusters whose activity was highly and jointly significant in both the neutral and sad recall conditions (p **_FDR_** *<*0.05). These SPM activity clusters were converted to ROIs using the MarsBaR region of interest (ROI) toolbox (http://marsbar.sourceforge.net). Our primary seed ROIs were located in the dorsal anterior cingulate cortex (‘paralimbic’ RSN; x, y, z = 3, −3, 51; 260 voxels) and the posterior cingulate cortex (‘default mode’ RSN; x, y, z = −3, −69, 21; 367 voxels), respectively. Our primary target ROIs were located in the right anterior insula cortex (‘paralimbic’ RSN; x, y, z = 48, 18, −12; 145 voxels) and the medial frontal cortex (‘default mode’ RSN; x, y, z = −9, 51, 36; 1062 voxels), respectively.

Initially, reference time courses were extracted from the neutral and sad recall condition scans for each subject, for each seed ROI. These time courses were calculated as the average time courses across all voxels inside each ROIs, which were then entered into first-level (single-subject) whole-brain, linear regression analyses in SPM5. To minimize the effect of global drift, voxel intensities were proportionally scaled by dividing each time point's value by the mean value of the whole-brain image at that time point. A high-pass filter set at 120 s was used to remove low frequency drifts below ∼0.008 Hz. Data were corrected for first-order serial autocorrelations using the AR(1) model in SPM5. Contrast images were generated for each subject by estimating the regression coefficient between each voxel and the reference time-series. These contrast images were then included in second-level (group) random-effects analyses using one-sample t tests. Resulting z transformed (Gaussianized) SPMs were thresholded at p **_FDR_** *<*0.05 (corrected) and represent the strength of functional connectivity from each seed to target ROI respectively (see Figure 5). Differences between conditions were considered significant at p<0.05 **_FDR_** after small volume correction (target ROI volume).

## Supporting Information

Figure S1 Visual and Sensorimotor Resting-State Networks. Statistical maps of two additional RSNs that were identified for the neutral condition alone (left panel; F & G). The middle and right panels display their varied reproducibility in each of the split-half analyses for the neutral recall (middle panel) and sad recall (right panel) conditions. All images are presented on a high-resolution single-subject MRI in standard neuroanatomical space (Montreal Neurological Institute, Colin-27). Corresponding color bars indicate the z score ranges of the displayed maps. Images are displayed in neurological convention (left = left).
(0.12 MB TIF)

Figure S2 Split-Half Analysis of Resting-State Networks. Assessment of the reproducibility of group ICA findings with the use of simple split-half analyses of the neutral and sad recall conditions. For both analyses, the split groups were assigned using a pseudo-random order. All images are presented on a high-resolution single-subject MRI in standard neuroanatomical space (Montreal Neurological Institute, Colin-27). Corresponding color bars indicate the z score ranges of the displayed maps. Images are displayed in neurological convention (left = left).
(0.27 MB TIF)

Figure S3 Cross-Correlation and Split-Half Analyses of the Sensorimotor and Visual Cortex Resting-State Networks. Regional functional connectivity maps of the visual cortical (F) and ‘sensorimotor’ (G) RSNs. Green clusters in the left most panel represent both of the seed ROIs. Both ROI types are overlaid on the regional functional connectivity maps derived from the cross-correlation analyses (CCAs). The middle and right panels show the reproducibility of the CCAs from a simple split-half analysis. All images are presented on a high-resolution single-subject MRI in standard neuroanatomical space (Montreal Neurological Institute, Colin-27). Corresponding color bars indicate the t score ranges of the displayed maps. Images are displayed in neurological convention (left = left).
(0.19 MB TIF)
