# Supplementary figures and images for: Modulation of Brain Resting-State Networks by Sad Mood Induction
Source: PLoS One. 2008 Mar 19;3(3):e1794. doi: 10.1371/journal.pone.0001794 (PMC2263138; doi:10.1371/journal.pone.0001794)

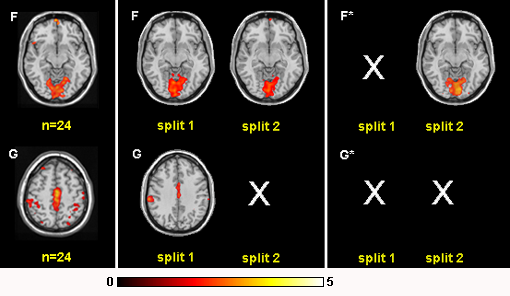

Supplement: Figure S1 — Visual and Sensorimotor Resting-State Networks. Statistical maps of two additional RSNs that were identified for the neutral condition alone (left panel; F & G). The middle and right panels display their varied reproducibility in each of the split-half analyses for the neutral recall (middle panel) and sad recall (right panel) conditions. All images are presented on a high-resolution single-subject MRI in standard neuroanatomical space (Montreal Neurological Institute, Colin-27). Corresponding color bars indicate the z score ranges of the displayed maps. Images are displayed in neurological convention (left = left). (0.12 MB TIF) [file pone.0001794.s001.tif]

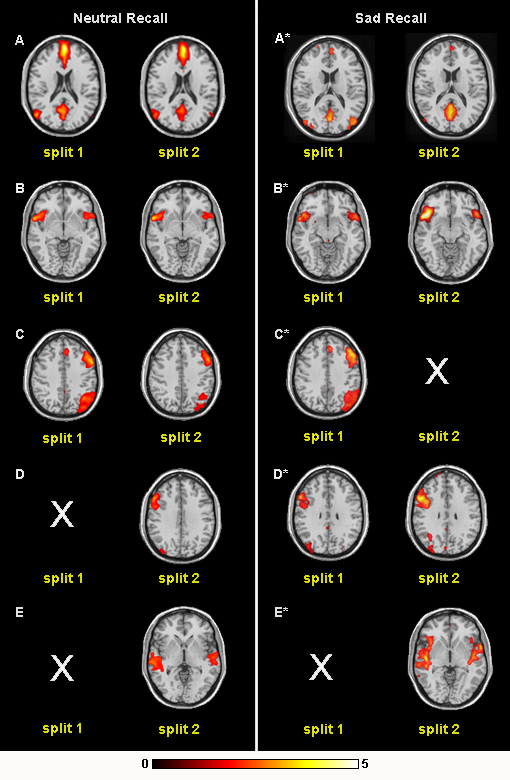

Supplement: Figure S2 — Split-Half Analysis of Resting-State Networks. Assessment of the reproducibility of group ICA findings with the use of simple split-half analyses of the neutral and sad recall conditions. For both analyses, the split groups were assigned using a pseudo-random order. All images are presented on a high-resolution single-subject MRI in standard neuroanatomical space (Montreal Neurological Institute, Colin-27). Corresponding color bars indicate the z score ranges of the displayed maps. Images are displayed in neurological convention (left = left). (0.27 MB TIF) [file pone.0001794.s002.tif]

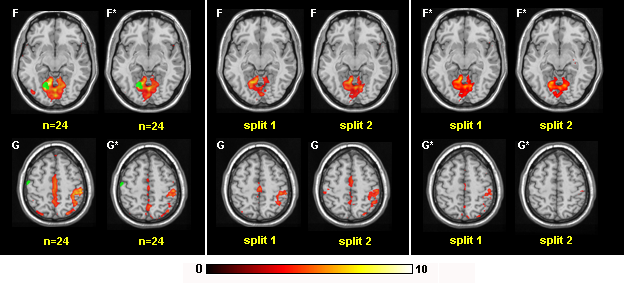

Supplement: Figure S3 — Cross-Correlation and Split-Half Analyses of the Sensorimotor and Visual Cortex Resting-State Networks. Regional functional connectivity maps of the visual cortical (F) and ‘sensorimotor’ (G) RSNs. Green clusters in the left most panel represent both of the seed ROIs. Both ROI types are overlaid on the regional functional connectivity maps derived from the cross-correlation analyses (CCAs). The middle and right panels show the reproducibility of the CCAs from a simple split-half analysis. All images are presented on a high-resolution single-subject MRI in standard neuroanatomical space (Montreal Neurological Institute, Colin-27). Corresponding color bars indicate the t score ranges of the displayed maps. Images are displayed in neurological convention (left = left). (0.19 MB TIF) [file pone.0001794.s003.tif]
